# Assessment of the psychometric properties of the traditional Chinese version of the cancer survivors’ self-efficacy scale

**DOI:** 10.1186/s41155-024-00317-y

**Published:** 2024-08-23

**Authors:** Ching-Hui Chien, Cheng-Keng Chuang, Chun-Te Wu, See-Tong Pang, Kuan-Lin Liu, Kai-Jie Yu

**Affiliations:** 1 College of Nursing, Peitou District, National Taipei University of Nursing and Health Sciences, No.365, Ming-Te Road, Taipei City, 112 Taiwan; 2 Division of Urology, Department of Surgery, Chang Gung Memorial Hospital at Linkou, Taoyuan City, Taiwan; 3 College of Medicine, Chang Gung University, Taoyuan, Taiwan; 4 Division of Urology, Department of Surgery, Chang Gung Memorial Hospital at Keelung, Keelung City, Taiwan

**Keywords:** Psychometrics, Reliability, Validity, Self-efficacy, Self-management, Cancer

## Abstract

**Background:**

The reliability and validity of the traditional Chinese version of the Cancer Survivors’ Self-Efficacy Scale (CS-SES-TC) has not been assessed.

**Objective:**

To assess the psychometric properties of the Traditional Chinese version of the CS-SES-TC.

**Methods:**

Participants were recruited from the outpatient departments of a hospital in Taiwan. A single questionnaire was administered to 300 genitourinary cancer survivors. The scales included in the initial questionnaire were the CS-SES-TC, the General Self-Efficacy Scale, the Center for Epidemiologic Studies Depression Scale (CES-D), and the Functional Assessment of Cancer Therapy-General scale (FACT-G). Data obtained from 300 survivors were used to confirm the structure through confirmatory factor analysis (CFA).

**Results:**

The CFA results indicate that the 11-item CS-SES-TC is consistent with the original scale. Furthermore, it was identified as a unidimensional scale, with the model showing acceptable goodness-of-fit (CFI = 0.99, TLI = 0.97). The factor loading of each item in the CS-SES-TC was above 0.6 and had convergent validity. Based on multiple-group CFA testing, the change (ΔCFI) between the unconstrained and constrained models was ≤ 0.01, indicating that measurement invariance holds for gender. The participants’ CS-SES-TC scores were positively correlated with their FACT-G scores and negatively correlated with their CES-D scores. The scales exhibited concurrent validity and discriminant validity. The CS-SES-TC had a Cronbach’s α in the range of .97–.98.

**Conclusion:**

The CS-SES-TC had acceptable reliability and validity. Healthcare workers can use this scale for ongoing assessment of the cancer-related self-efficacy of cancer survivors.

## Introduction

In 2022, Approximately 20.0 million people worldwide received new diagnoses of cancer, and about 9.7 million people died of cancer (Bray et al., 2024). In Taiwan, 121,762 people received cancer diagnoses, and 51,656 people died of cancer in 2021 (Health Promotion Administration, Ministry of Health and Welfare, 2023). As medical technology advances, the survival rate of patients with cancer has improved. Nevertheless, the diagnosis and treatment of cancer causes physical, psychological, and social changes at various levels in cancer survivors and can induce cancer-related fatigue, cognitive disorders (Joly et al., 2019), urinary and fecal incontinence (Ramaseshan et al., 2018; Schiffmann et al., 2020), anxiety, depression, fear of cancer recurrence (Yi & Syrjala, 2017), and social isolation (van Roij et al., 2019). Therefore, cancer survivors must manage their cancer-related health problems to maintain a satisfying life (Marzorati et al., 2017).

Self-management, which entails individuals engaging in self-care activities to maintain a healthy lifestyle, evaluate and monitor symptoms, and respond to the effects of health-related crises with the assistance of medical personnel, is a continuous process (Lorig & Holman, 2003). Self-management programs can help alleviate the physical symptoms and psychological distress of cancer survivors (Agbejule et al., 2022; Faithfull et al., 2011; Howell et al., 2017; Somers et al., 2015). Scholars have proposed a conceptual framework for the recovery of health and well-being for cancer survivors. According to this conceptual framework, an individual’s interactions with the environment will affect their cancer-related self-efficacy. Cancer-related self-efficacy is the degree of confidence (self-efficacy) of cancer survivors in managing cancer- and treatment-related health problems after primary treatment. Cancer-related self-efficacy affects the strategies that an individual adopts in the self-management of cancer- and treatment-related health problems. These strategies are ultimately associated with the recovery of an individual’s health and well-being (Foster & Fenlon, 2011; Foster et al., 2015).

The Cancer Survivors’ Self-Efficacy Scale (CS-SES) has been commonly used to measure cancer survivors’ self-efficacy in managing cancer- and treatment-related health problems (Chien et al., 2023, 2022b; Foster et al., 2015, 2016; Grimmett et al., 2017; Liu et al., 2022; Nelson et al., 2022). The CS-SES was developed by modifying the Self-Efficacy for Managing Chronic Disease 6-Item Scale (SEMCD, Lorig et al., 2001a, 2001b), specifically by adding five new items. The SEMCD scale consists of six items assessing confidence in managing illness or health-related issues. The items, in sequence, are fatigue, physical discomfort or pain, emotional distress, symptoms or health problems, and doing things other than taking medication and performing tasks and activities (Lorig et al., 2001a, 2001b). Based on interviews with 30 cancer survivors (Foster & Fenlon, 2011), the developers of CS-SES modified items one to three and five to six of the SEMCD to make the scale suitable for cancer survivors and added five new items, resulting in the CS-SES (Foster et al., 2013). The CS-SES is a unidimensional scale. In the development of the scale, nonparametric item response theory was used to determine the scale's dimension and evaluate the scalability of the new items added to the original scale (Foster et al., 2013). The CS-SES has been translated into Korean (Kim et al., 2019) and Traditional Chinese (Chien et al., 2023, 2022b; Liu et al., 2022).

The CS-SES was previously translated into Korean (Kim et al., 2019). Item six of the original scale (regarding self-efficacy to do things other than just taking medication) was deleted from the Korean version, primarily due to factor loading being below 0.5 and cancer survivors not being required to take medication on a daily basis. In addition, Items 5 and 6 (regarding self-efficacy to complete different tasks and activities) were partially redundant. According to the results of exploratory factor analysis (EFA), the 10-item Korean version of the CS-SES contains two subscales, namely the self-efficacy for managing health problems and self-efficacy for seeking help and support. The internal consistency of the Korean version of CS-SES, the subscale of self-efficacy for managing health problems, and the subscale of self-efficacy for seeking help and support had Cronbach’s α values of 0.92, 0.86, and 0.92, respectively (Kim et al., 2019).

The CS-SES was previously translated into Traditional Chinese in a study on prostate cancer using both forward and backward translation methods (Liu et al., 2022) and has been applied in research on prostate (Chien et al., 2023, 2022b) and kidney cancer (Liu et al., 2022). However, the reliability and validity of the Traditional Chinese version of the CS-SES (CS-SES-TC) has not been assessed. Therefore, this study was conducted to assess the reliability and validity of the CS-SES-TC.

## Methods

### Research design and participants

Based on sample accessibility, participants were recruited from a urology and cancer center outpatient department of a medical center in Taiwan from April 6, 2020, to December 26, 2020. Cancer survivors who met the recruitment criteria and agreed to participate in the study completed the questionnaires after signing the consent form. To evaluate the 2-week test–retest reliability of the scale, some of the participants were invited to complete the CS-SES-TC again after 2 weeks.

The inclusion criteria were as follows: (a) diagnosis of genitourinary cancer by a physician; (b) completion of primary treatment; (c) an age of over 20 years; (d) ability to communicate; (e) no history of depression, anxiety, bipolar disorder, or dementia; and (f) a score of 2 points or lower on the Eastern Cooperative Oncology Group performance status scale.

The CS-SES-TC contains 11 items. According to relevant literature, the sample size for factor analysis should be three to 20 times the number of items (Mundfrom et al., 2005). Therefore, the sample size for the confirmatory factor analysis (CFA) was estimated to be 300 participants. 50 of these participants were invited to complete the CS-SES-TC again after a two-week interval (Park et al., 2018) to evaluate the test–retest reliability of the scale.

During the study period, 680 cancer survivors were identified as potential participants; of these, 544 cases met the recruitment criteria after evaluation. A total of 370 cancer survivors were invited to participate in this study, 70 of whom refused to participate due to lack of interest (*n* = 41), time constraints (*n* = 22), and refusal from family members (*n* = 7). In total, 300 people completed the first questionnaire survey. Fifty of the participants were invited to complete a second CS-SES, 49 of whom actually completed the scale.

### Recruitment procedure

Cancer survivors who potentially met the recruitment criteria were referred to the study by urologists. The research assistant evaluated each individual's eligibility according to the inclusion and exclusion criteria. After an eligible participant provided informed consent and signed the consent form, they completed the questionnaire in a private space. While the participant completed the questionnaire, the research assistant waited nearby to help and provide an explanation if required. After completing the first questionnaire, the participants willing to retake the CS-SES-TC in 2 weeks were given the questionnaire and a reply envelope. A text message (SMS) was sent out as a reminder to complete the survey 2 weeks later.

### Measures

#### Demographics and disease attributes

We collected information on each participant’s age, gender, religious beliefs, education level, marital status, occupational status, exercise habits, cancer type, cancer stage, cancer treatment methods, and months since diagnosis.

#### CS-SES

The CS-SES-TC was used to measure the self-confidence (cancer-related self-efficacy) of cancer survivors in self-managing cancer- and treatment-related health problems (Foster et al., 2013, 2015). The CS-SES was translated from English into Traditional Chinese after obtaining permission from the original developer and has been used in studies of prostate (Chien et al., 2023, 2022b and kidney cancer survivors (Liu et al., 2022). The scale contains 11 items, with scores ranging from 1 to 10 points. Lower scores indicate lower cancer-related self-efficacy: a score of 1 point signifies a complete lack of self-efficacy, and a score of 10 indicates complete self-efficacy (Foster et al., 2013, 2015). Regarding internal reliability, the scale has a Cronbach’s α of 0.92 (Foster et al., 2013).

#### General self-efficacy scale

The General Self-Efficacy Scale (GSE; Jerusalem & Schwarzer, 1992; Zhang & Schwarzer, 1995) was the criterion tool used in this study. The scale has 10 items, with total scores ranging from 1 to 4 points. Lower scores indicate lower self-efficacy. The scale has construct validity, a Cronbach’s α of 0.91–0.92 (Cheung & Sun, 1999; Zhang & Schwarzer, 1995), and a test–retest reliability (*r*) of 0.70 (Cheung & Sun, 1999). The scale has been used to measure the general self-efficacy of patients with cancer (Chien et al., 2022a; Liang et al., 2015; Wu et al., 2021).

#### Center for epidemiologic studies depression scale

The Chinese version of the Center for Epidemiologic Studies Depression Scale (CES-D) contains a total of 20 items, each of which is scored from 0 to 3. A score of 0 indicates that an individual “rarely” experiences a given depressive symptom (less than 1 day a week), and a score of 3 indicates that they “always” experience that symptom (more than 5 days a week; Chien & Cheng, 1985; Radloff, 1977). Individuals with scores of 0–15 are considered to not have depression, and individuals with scores of 16–60 are considered to have depression (Chien & Cheng, 1985). The scale has high reliability and validity (Chien & Cheng, 1985; Radloff, 1977), and the Traditional Chinese version has been widely used to measure depression in patients with cancer (Fang et al., 2015; Zhao et al., 2021).

#### Functional assessment of cancer therapy–general

The 27-item Traditional Chinese version of the Functional Assessment of Cancer Therapy–General (FACT-G) scale is used to evaluate the cancer-specific quality of life in cancer survivors (Cella et al., 1993; Cheung et al., 2009). The total scale comprises four domains: physical well-being, functional well-being, social/familial well-being, and emotional well-being. The scale employs a 5-point scoring method, with higher scores indicating better quality of life (Cheung et al., 2009). The scale exhibits adequate reliability and validity (Cella et al., 1993; Cheung et al., 2009).

### Statistical methods

IBM SPSS (Statistical Package for the Social Sciences) version 22 and AMOS (Analysis of Moment Structures) version 18 were used for data analysis. IBM SPSS software was used for the following: descriptive statistics (means, standard deviation, range, quartile, and percentages), Pearson product-moment correlation, independent sample *t-test*, corrected item-total correlation, Cronbach’s α (internal consistency), and intraclass correlation coefficient (ICC). AMOS software was used for CFA. A two-tailed test was used, with the significance level set at *p* < 0.05.

Data obtained from 300 cancer survivors were used for CFA, using the maximum likelihood method, to validate the structure of the CS-SES-TC and evaluate the scale's convergent validity. Additionally, multiple-group CFA was employed to test measurement invariance across genders. The model goodness-of-fit criteria were as follows: a comparative fit index (CFI) ≥ 0.95, a Tucker–Lewis index (TLI) ≥ 0.95, a goodness-of-fit index (GFI) ≥ 0.90, a standardized root mean square residual (S-RMR) < 0.05, and a root mean square error of approximation (RMSEA) < 0.1 (Bowen & Guo, 2012; Fadlelmula, 2011). When the goodness-of-fit did not meet these criteria, we referred to the modification index (MI) value. We corrected the correlation of the measurement error of each item (covariance) to improve the goodness-of-fit of the model (Hoyle, 1995). For the measurement of invariance testing by gender, a value of ∆CFI ≤ 0.01 between the unconstrained model and constrained model indicates measurement invariance across genders (Cheung & Rensvold, 2002; Tan & Pektaş, 2020).

After confirming the structure of the CS-SES-TC, we used all the participants’ data to assess the scale's item analysis, criterion validity, concurrent validity, discriminant validity, internal consistency, and test–retest reliability.

### Research ethics

Recruitment of participants began after the approval of the study by the human research ethics committee of the receiving hospital. During the research process, the researchers abided by the research code of ethics, respected the autonomy of the participants, and obtained informed consent and a signed consent form from each participant. The participants could withdraw from the study without affecting their original rights to treatment. The research team maintained the privacy of participants. Participants were assured that the data collected would be used for academic purposes only and would be unidentifiable when published.

## Results

The mean age of the participants was 63.1 years. In total, 83.3% of the participants were men, and 85.7% were married or cohabitating. Almost all the participants (98.3%) had a formal education, and 67.6% were unemployed or retired. Most (70%) of the participants reported that they exercised regularly. The mean time since diagnosis was 61.1 months. Regarding cancer type, 32.3% of the participants had prostate cancer, and 33.0% had kidney cancer. A total of 66.6% of the participants had stage 0 to II cancer, and 68.7% of the participants had undergone surgery only (Table 1).

**Table 1:** Demographic and disease characteristics of cancer survivors (*n* = 300)

Variable	*n* (%)	Mean ± SD	Range
Age in years		63.1 ± 11.9	22–87 (Q1 = 57.00, Q2 = 66.00, Q3 = 71.00, Q4 = 87.00)
Gender			
Male	250 (83.3)		
Female	50 (16.7)		
Marital status			
Single	16 (5.3)		
Married/Cohabitating	257 (85.7)		
Widowed	18 (6.0)		
Divorced	9 (3.0)		
Educational level			
None	5 (1.7)		
Elementary/Junior high school	98 (32.7)		
High school	92 (30.7)		
College/University	90 (30.0)		
Post-graduate	15 (5.0)		
Occupational status			
None	31 (10.3)		
Retired	172 (57.3)		
Employed	96 (32.0)		
Regular exercise			
No	90 (30.0)		
Yes	210 (70.0)		
Months since diagnosis		61.6 ± 43.1	1.5–200.2 (Q1 = 26.60, Q2 = 54.83, Q3 = 89.30, Q4 = 200.2)
Cancer type			
Prostate cancer	97 (32.3)		
Kidney cancer	99 (33.0)		
Ureteral cancer	9 (3.0)		
Bladder cancer	69 (23.0)		
Urethral cancer	3 (1.0)		
Testicular cancer	7 (2.3)		
Bladder and kidney cancer	6 (2.0)		
Bladder and ureteral cancer	8 (2.7)		
Prostate and kidney cancer	2 (0.7)		
Cancer stage			
Stage 0/I	118 (39.3)		
Stage II	82 (27.3)		
Stage III	51 (17.0)		
Stage IV	15 (5.0)		
Unknown	34 (11.4)		
Primary treatment			
Surgery	206 (68.7)		
Radiation therapy	13 (4.3)		
Chemotherapy	1 (0.3)		
Surgery and intravesical therapy	56 (18.6)		
Other	24 (8.1)		

### Structural confirmation and convergent validity of the CS-SES-TC

CFA was conducted using data obtained from 300 participants. Unidimensional analysis for the 11 items of the scale were set based on the original structure of the scale. The CFA produced a Kaiser–Meyer–Olkin index of 0.92, and the Bartlett’s test of sphericity result was 4562.68 (*p* < 0.001). The results indicated that the factor loading of each item was above 0.6 (ranging from 0.64 to 0.95), which could explain 74.50% of the variance (Table 2). The model had an acceptable goodness-of-fit (chi-square = 110.36, degrees of freedom = 30, *p* < 0.001, CFI = 0.99, TLI = 0.97, S-RMR = 0.04, RMSEA = 0.089, GFI = 0.94) after adjusting for the correlation among 14 residual errors in sequence according to the MI. The scale exhibited convergent validity.

**Table 2:** Factor loading results and item analysis of the scale (*n* = 300)

Item	Mean (SD)	Corrected item-total correlation	Highest- and lowest-score groups (*t*-score)	Factor loading from CFA	Factor loading from multiple-group CFA	
					Male	Female
1. Keep the fatigue from interfering with things	7.66 (1.62)	0.88	-22.15^***^	0.92	0.89	0.93
2. Keep the physical discomfort or pain from interfering with things	7.59 (1.73)	0.87	-25.33^***^	0.93	0.92	0.86
3. Keep the emotional distress from interfering with things	7.53 (1.71)	0.93	-23.19^***^	0.91	0.89	0.87
4. Keep any other symptoms or health problems from interfering with things	7.54 (1.66)	0.92	-22.10^***^	0.93	0.94	0.98
5. Do different tasks and activities	7.66 (1.62)	0.87	-24.77^***^	0.95	0.94	0.95
6. Do things other than just taking medication	7.69 (1.57)	0.83	-23.55^***^	0.95	0.95	0.92
7. Access information	7.99 (1.52)	0.84	-18.24^***^	0.78	0.78	0.80
8. Access people to help and support you	8.00 (1.60)	0.91	-15.60^***^	0.70	0.67	0.80
9. Deal with the problems	7.78 (1.71)	0.91	-19.25^***^	0.83	0.83	0.74
10. Contact doctor	8.49 (1.43)	0.85	-15.04^***^	0.64	0.61	0.70
11. Get support for the problems from professionals	8.39 (1.47)	0.85	-15.00^***^	0.65	0.61	0.72
Total score	86.25 (15.20)		-30.35^***^			
Eigenvalue				8.19		
Variance explained (%)				74.50		

*CFA* Confirmatory factor analysis, *SD* Standard deviation

^***^p < .001

### Measurement invariance across gender

Further testing of the measurement invariance of CS-SES-TC by gender showed that factor loading for males and females was above 0.6 for each item, and all models had acceptable fit indices (see Tables 2 and 3). Compared to the unconstrained model (CFI = 0.970), the CFIs of the remaining constrained models were similar to that of the unconstrained model, with differences not exceeding 0.01 (measurement weights model = 0.971; structural covariances model = 0.972; measurement residuals model = 0.960). This indicated that the measurement model exhibited configural, measurement, and structural invariance across genders.

**Table 3:** Results of the measurement invariance test by gender (*n* = 300)

Model	*χ*^*2*^	*df*	*χ*^*2*^*/df*	CFI	∆CFI	RMSEA	GFI	TLI
Unconstrained	199.65	60	3.33	0.970		0.09	0.90	0.95
Measurement weights	203.78	70	2.91	0.971	-0.001	0.08	0.90	0.96
Structural covariances	203.79	71	2.87	0.972	-0.002	0.08	0.90	0.96
Measurement residuals	285..41	96	2.97	0.960	0.010	0.08	0.86	0.95

*CFI* Comparative Fit Index, *GFI* Goodness-of-Fit Index, *RMSEA* Root Mean Square Error of Approximation, *TLI* Tucker–Lewis index

### Item analysis and ceiling and floor effects

The mean CS-SES-TC score of the participants was 86.25 (Standard Deviation, SD = 15.20). The participants with the highest 27% of CS-SES-TC scores were categorized as the high-score group, whereas those with the lowest 27% were categorized as the low-score group. The scores of the two groups, both on the overall scale and for individual items, differed significantly (*p* < 0.001, Table 2).

### Criterion validity of the CS-SES-TC

A significant correlation was identified between the participants’ CS-SES-TC and GSE scores (*r* = 0.52; *p* < 0.001; Table 4). Therefore, the scale exhibited criterion validity.

**Table 4:** Correlations between scale scores

Scale/Variable	Cancer Survivors’ Self-Efficacy Scale- Traditional Chinese version			
	*n*	Mean (SD)	*r/t*	*p*
General Self-Efficacy Scale	300	30.6 (5.2)	0.52^***^	< 0.001
FACT-G Scale	300	88.19 (11.43)	0.37^***^	< 0.001
Physical well-being	300	25.39 (3.74)	0.19^***^	< 0.001
Social/family well-being	300	20.90 (4.37)	0.33^***^	< 0.001
Emotional well-being	300	21.74 (3.05)	0.29^***^	< 0.001
Functional well-being	300	21.67 (4.93)	0.29^***^	< 0.001
CES-D Scale	300	7.41 (7.2)	-0.31^***^	< 0.001
Depression			2.79^**^	0.006
Non-depressed case (CES-D 1–15 points)	264	87.15 (14.81)		
Depressed case (CES-D ≥ 16 points)	36	79.69 (16.62)		
Regular exercise			-2.22^*^	0.027
No	90	83.30 (16.48)		
Yes	210	87.52 (14.1)		

*CES-D* Center for Epidemiologic Studies Depression Scale, *FACT-G* Functional Assessment of Cancer Therapy–General

^*^p < 0.05

^**^p < .01

^***^p < .001

### Concurrent and discriminant validity of the CS-SES-TC

The CS-SES-TC scores of the participants were significantly and positively correlated with their scores on the FACT-G (*r* = 0.37, *p* < 0.001) and each of its subdimensions (physical well-being: *r* = 0.19, *p* < 0.001; social/family well-being: *r* = 0.33, *p* < 0.001; emotional well-being: *r* = 0.29, *p* < 0.001; functional well-being: *r* = 0.29, *p* < 0.001).

The participants’ CS-SES-TC scores were also significantly and negatively correlated with their CES-D scores (*r* =  − 0.31, *p* < 0.001). When the participants were divided into depressed and nondepressed groups according to their total CES-D scores, the participants with depression had a lower average CS-SES-TC score than those without depression (*t* = 2.79, *p* = 0.006).

Furthermore, the average CS-SES-TC score of the participants who reported not exercising regularly was lower than that of those who reported exercising regularly (*t* =  − 2.22, *p* = 0.027). According to these data, the CS-SES-TC exhibits concurrent and discriminant validity (Table 4).

### Internal consistency reliability and test–retest reliability of the CS-SES-TC

Regarding internal consistency reliability, the scale had a Cronbach’s ɑ of 0.97 for all participants (*n* = 300) and 0.98 for the subsample used for the test–retest analysis (*n* = 49). For the two-week test–retest reliability, the scale had an estimated ICC value of 0.76.

## Discussion

The results of this study support the hypothesis that the CS-SES-TC has acceptable reliability and validity. According to the CFA performed here, and in contrast to the Korean version of the scale (Kim et al., 2019), both the CS-SES-TC and original scale are unidimensional. In addition, the factor loading of item 6 was 0.95.

The 10-item Korean version of the CS-SES exhibited adequate model goodness of fit (Kim et al., 2019). In this study, according to the CFA, the initial model goodness of fit was suboptimal, but after adjusting for MI values, the GFI reached a value within the acceptable range. In addition, the correlation between the CS-SES-TC score and the criterion tool in this study was 0.52, which supports the criterion validity of the scale. This correlation was similar to those of the Korean version of the CS-SES and the GSE (*r* = 0.51; Kim et al., 2019) but was higher than the correlations between other cancer-related self-efficacy scales and the GSE (0.40–0.47; Huang et al., 2017; Liang et al., 2015).

The internal consistency reliability of the CS-SES-TC obtained in this study was between 0.97 and 0.98 (all above 0.7), indicating that the scale had satisfactory internal consistency reliability (Taber, 2018). This value was similar to those of the original scale (0.92; Foster et al., 2013) and the 10-item Korean version of the scale (0.92; Kim et al., 2019). When the internal consistency of a scale is too high, some items on the scale can be shortened (Taber, 2018). Therefore, researchers may consider developing a shorter version of the scale in the future. In addition, the ICC in this study was 0.76, indicating that the scale has adequate test–retest reliability (Koo & Li, 2016). The test–retest reliability of the original scale (Foster et al., 2013) and that of the 10-item Korean version of the scale (Kim et al., 2019) have not been measured.

This study established measurement invariance for the CS-SES-TC across genders, ensuring that the observed differences in CS-SES-TC among genders reflect genuine differences in cancer-related self-efficacy. The CS-SES-TC can be used to evaluate the cancer-related self-efficacy of Chinese-speaking cancer survivors in managing disease- and treatment-related health problems to enable healthcare workers to provide appropriate care promptly, thereby further improving the survivors’ cancer-related self-efficacy. According to the relevant theories, improving cancer survivors’ self-efficacy helps them engage in self-management activities, such as exercise (Bandura, 1977), restore their own health and well-being (Foster & Fenlon, 2011), and even achieve personal growth (Brennan, 2001).

## Limitations

A strength of this study is its use of CFA to evaluate the structure of the CS-SES-TC, utilizing data from participants with a Chinese cultural background, which is consistent with the original scale. Additionally, the study used CFA to evaluate the measurement invariance of the CS-SES-TC across genders and assessed the test–retest reliability of the scale to ensure its stability.

However, the study has some limitations. Participants were recruited from a 3,700 bed medical center in Taiwan, and all of these patients had genitourinary cancer. Future studies could use larger sample sizes across multiple centers and include survivors of different types of cancer and various ethnic groups to evaluate the psychometric properties of the CS-SES-TC further. Moreover, incorporating a more geographically and culturally diverse sample could enhance the generalizability of the findings.

## Conclusion

The results of this study indicate that the 11-item Chinese version of the CS-SES is consistent with the original scale, both of which were identified as unidimensional scales. The Chinese version of the scale exhibits acceptable convergent validity, discriminant validity, concurrent validity, internal consistency reliability, and test–retest reliability. The scale can be used to assess the self-efficacy of Chinese-speaking cancer survivors in managing problems caused by cancer and its treatment. Health-care workers can provide prompt assistance to promote self-management further and improve cancer survivors' well-being and quality of life.

## Data Availability

The data used in this study are stored and managed by the corresponding author, to whom readers can direct any questions. The data are not publicly available due to the consideration of ethics, the researchers shall maintain the privacy of the participants, and research data should be used only for academic.

## Abbreviations

AMOS: Analysis of Moment Structures
CFA: Confirmatory factor analysis
CFI: Comparative Fit Index
CI: Confidence Interval
CS-SES: Cancer Survivors’ Self-Efficacy Scale
CS-SES-TC: Traditional Chinese version of the CS-SES
EFA: Exploratory factor analysis
FACT-G: Functional Assessment of Cancer Therapy–General
GFI: Goodness-of-Fit Index
GSE: General Self-Efficacy Scale
MI: Modification Index
TLI: Tucker–Lewis index
*r*: Correlation coefficient
RMSEA: Root Mean Square Error of Approximation
SD: Standard Deviation
SPSS: Statistical Package for the Social Sciences
S-RMR: Standardized Root Mean Square Residual
